# Clinical-grade AI model for molecular subtyping of endometrial cancer: a multi-center cohort study in China

**DOI:** 10.1186/s43556-025-00341-z

**Published:** 2025-11-10

**Authors:** Peng Qi, Tianliang Yao, Hu Li, Jingnan Zhu, Jianye Li, Xuezhen Luo, Qizhi He, Yiran Li

**Affiliations:** 1 Department of Control Science and Engineering, College of Electronics and Information Engineering, Tongji University, Shanghai, 201210 China; 2 Centre for Assisted Reproduction, Shanghai Key Laboratory of Maternal-Fetal Medicine, Shanghai Institute of Maternal-Fetal Medicine and Gynecologic Oncology, Shanghai First Maternity and Infant Hospital, School of Medicine, Tongji University, Shanghai, 200092 China; 3 Pingdingshan Maternal and Child Health Hospital, Henan, 467000 China; 4 Department of Gynecology, Obstetrics and Gynecology Hospital of Fudan University, Shanghai, 200011 China; 5 Department of Pathology, Shanghai First Maternity and Infant Hospital, School of Medicine, Tongji University, Shanghai, 200092 China; 6 State Key Laboratory of Cardiovascular Diseases and Medical Innovation Center, Shanghai East Hospital, School of Medicine, Tongji University, Shanghai, 200092 China

**Keywords:** Endometrial cancer, Molecular subtyping, Whole slide imaging, Deep learning, Artificial intelligence, Digital pathology

## Abstract

Accurate molecular subtyping is essential for guiding precision treatment and prognostic stratification in endometrial cancer (EC). However, current methods, based on Sanger sequencing and immunohistochemistry (IHC), are costly, time-intensive, and difficult to implement widely in routine clinical practice, particularly in resource-limited settings. To overcome these challenges, we developed a deep-learning pipeline that directly infers EC molecular subtypes from routine hematoxylin-and-eosin (H&E) whole-slide images (WSIs). The framework integrates super-resolution enhancement (SRResGAN), transformer-based lesion segmentation (MedSAM), and a ResNet-101 classifier for molecular subtype prediction, with an LSTM module for survival modeling. This retrospective study included 393 Chinese patients diagnosed between 2010 and 2018, all with ≥ 5 years of follow-up. Molecular subtypes—POLE^mut^, mismatch repair-deficient (MMRd), p53abnormal (p53abn), and no specific molecular profile (NSMP)—were confirmed by Sanger sequencing and immunohistochemistry. The model achieved high classification accuracies (92% for POLE^mut^ and MMRd, 91% for p53abn, and 90% for NSMP), with a strong correlation between predicted and observed survival (R2 = 0.9692; MAE = 123 days). External validation on two independent cohorts (*N* = 35 and *N* = 83) confirmed robust generalizability across institutions. This study represents the first large-scale, multicenter, AI-based digital pathology model for EC molecular classification in China. The proposed workflow provides an automated, interpretable, and cost-efficient alternative to conventional molecular testing, supporting precision oncology, fertility-preserving management, and clinical decision-making in real-world practice.

**Supplementary Information:**

The online version contains supplementary material available at 10.1186/s43556-025-00341-z.

## Introduction

Endometrial cancer (EC) is the second most common malignancy of the female reproductive system in China, with steadily increasing incidence and mortality in recent years. This rise has been accompanied by a noticeable shift toward earlier onset, with a growing proportion of patients diagnosed during reproductive age [1, 2]. These epidemiologic changes complicate clinical management and highlight the need for accurate diagnosis and individualized treatment planning, particularly for fertility-preserving candidates.

The rising incidence of EC has further strained the already limited pool of experienced pathologists, exacerbating diagnostic workloads and variability [3]. Accurate molecular subtyping is essential for prognostic evaluation and the selection of appropriate therapy. Advances in molecular oncology have refined the Cancer Genome Atlas (TCGA) classification, which allows improved stratification of tumors with distinct clinical behavior and molecular profiles, providing a framework for more individualized patient management [4]. However, conventional assays based on Sanger sequencing and immunohistochemistry (IHC) remain costly, labor-intensive, and time-consuming, limiting their scalability in routine diagnostic workflows. This practical limitation underscores the urgent demand for more efficient, accessible, and reproducible methods for EC molecular classification.

Digital pathology has become an integral component of modern diagnostic workflows due to rapid developments in artificial intelligence (AI) [5, 6]. Deep learning models have achieved promising results in the automated analysis of whole slide images (WSIs) for various cancers, including lung carcinoma [7], by identifying complex morphological features even in sparsely cellular regions [8]. In other tumor types, AI and deep learning have also shown strong potential in molecular subtyping, prognostication, and treatment stratification. For example, in bladder cancer, Zheng et al. developed a pathology-based deep learning model that accurately distinguished basal and luminal subtypes from hematoxylin and eosin (H&E) slides, achieving area-under-the-curve (AUC) values of approximately 0.88 in internal validation and moderate performance in external cohorts [9]. Moreover, a recent meta-analysis demonstrated that AI-assisted digital pathology achieves high pooled sensitivity and specificity across multiple cancer types, supporting its diagnostic reliability [10]. The 2023 International Federation of Gynecology and Obstetrics (FIGO) guidelines further underscored the clinical importance of molecular classification in EC [11]. Recent studies have explored AI-based models for molecular subtype prediction using WSIs in recent multi-center datasets, demonstrating that deep learning can extract subtype-specific morphological characteristics and contribute to prognostic assessment [12, 13]. However, most of these studies have predominantly been on Western datasets, limiting the applicability of their findings to Chinese populations.

In this context, there is an urgent need for an efficient, clinically viable, and cost-effective strategy for molecular subtyping of EC in China. To address this issue, a cohort of 444 patients newly diagnosed with EC between 2010 and 2018 was retrospectively assembled. Each patient underwent POLE mutation testing, immunohistochemistry (IHC) evaluation, and long-term follow-up. WSIs were generated from diagnostic slides using Digital Slide Assistant Lite, and relevant clinical data were collected.

The deep learning model was trained to predict molecular subtypes defined by IHC and Sanger sequencing, which served as the reference standard. This study aimed to develop and validate a WSI-based molecular subtyping pipeline tailored to Chinese patients. Model architectures were systematically compared for their ability to classify the four TCGA subtypes, POLE-mutated (POLE^mut^), mismatch repair-deficient (MMRd), p53abnormal (p53abn), and no specific molecular profile (NSMP), and to predict survival outcomes. Rather than focusing only on technical novelty, this work prioritizes clinical applicability, scalability, and generalizability across Chinese centers, supporting the advancement of precision oncology in EC management.

## Results

### Study workflow and baseline characteristics

**Fig. 1:**
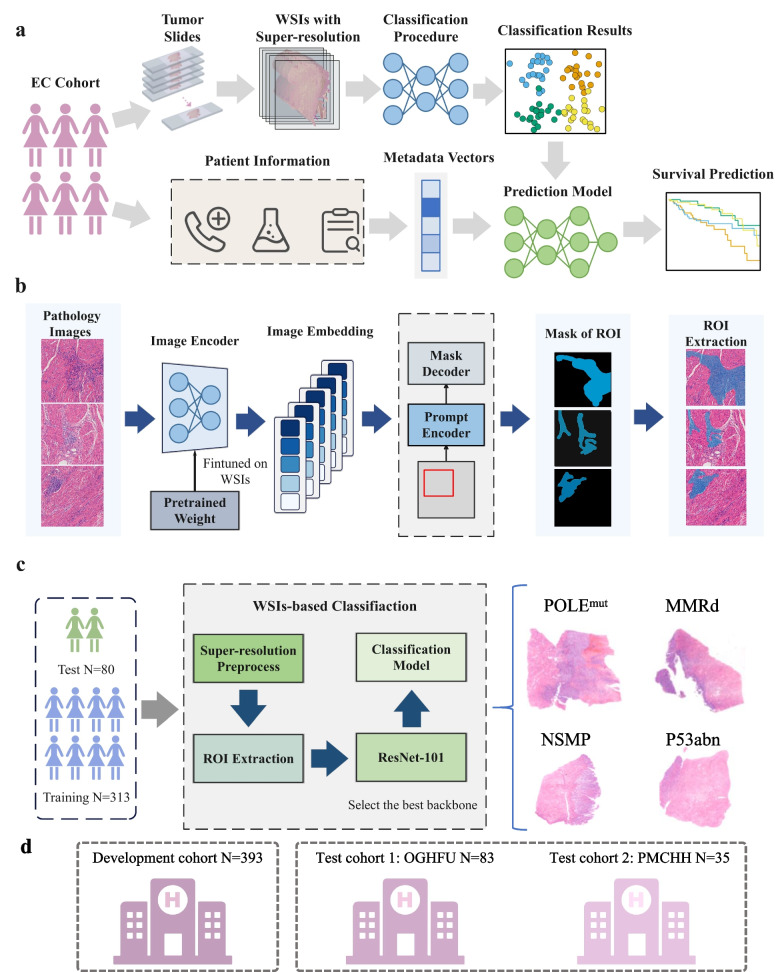
Overview of the study design and processing pipeline. **a** Overall study design. Tumor slides from the EC cohort were digitized into WSIs, preprocessed with SRResGAN for super-resolution and MedSAM for ROI extraction. The dataset was split into training, validation, and test sets with balanced sampling. Multiple backbones were compared, and ResNet-101 was selected for final classification. **b** Processing pipeline. Low-resolution WSIs were enhanced using SRResGAN, followed by ROI segmentation with a fine-tuned MedSAM model. Extracted high-resolution ROIs were classified into molecular subtypes using the EC model. **c** ROI segmentation and subtype classification. ROIs were classified into four TCGA molecular subtypes: POLEmut, MMRd, NSMP, and p53abn. Representative H&E images are shown. **d** Multi-center cohorts. The development cohort (*n* = 393) was used for training and internal validation, while two external cohorts from OGHFU (*n* = 83) and PMCHH (*n* = 35) were used for independent validation to assess generalizability

The overall pipeline of our study, including slide digitization, super-resolution enhancement, ROIs (regions of interest) segmentation, and subtype classification, is illustrated in Fig. 1.

A total of 444 patients diagnosed with EC between 2010 and 2018 were initially included in the study. After excluding 51 patients with incomplete clinical or pathological data, 393 patients were retained for analysis. At the time of the last follow-up, 315 patients (80.15%) were alive, 47 (11.96%) had died, and 31 (7.89%) were lost to follow-up. Molecular classification showed that the most frequent subtype was NSMP (40.71%), followed by p53abn (29.26%), MMRd (18.58%), and POLE^mut^ (11.45%). Most patients were 50–70 years old (66.41%), while 40–50 years old accounted for 25.19%, and the proportions of patients aged ≥ 70 and < 40 years were relatively low. Histologically, endometrioid carcinoma was the predominant subtype (84.99%), while serous carcinoma and carcinosarcoma accounted for 7.63% and 3.56%, respectively. According to the FIGO staging system, stage I was the most common (72.52%), while stage II and stage III each comprised approximately 13% of cases. Stage IV was rare (0.51%). Body mass index (BMI) analysis indicated that overweight and obesity were prevalent, together accounting for 75.31% of the cohort (Table 1). There were no statistically significant differences among molecular subtypes with respect to age distribution, histological subtype, or BMI. However, FIGO stage distribution differed significantly among the subtypes (*P* = 0.01), with POLE^mut^ tumors more frequently identified at stage I (Table S1).

**Table 1:** Baseline Characteristics of Patients

Characteristic	*N* = 393
Death, n(%)	
Alive	315 (80.15%)
Dead	47 (11.96%)
Lost	31 (7.89%)
Age group, n(%)	
20–40 years	18 (4.58%)
40–50 years	99 (25.19%)
50–70 years	261 (66.41%)
70 + years	15 (3.82%)
Molecular classification, n(%)	
MMRd	73 (18.58%)
NSMP	160 (40.71%)
p53abn	115 (29.26%)
POLE^mut^	45 (11.45%)
Histological type, n(%)	
Carcinosarcomas	14 (3.56%)
Clear Cell Carcinoma	5 (1.27%)
Endometrioid Carcinoma	334 (84.99%)
Mixed Carcinoma	9 (2.29%)
Serous Carcinoma	30 (7.63%)
Undifferentiated Carcinoma	1 (0.25%)
Stage, n(%)	
I	285 (72.52%)
II	52 (13.23%)
III	54 (13.74%)
IV	2 (0.51%)
BMI group, n(%)	
Under weight	12 (3.05%)
Normal weight	85 (21.63%)
Over weight	188 (47.83%)
Obese	108 (27.48%)

Overall, the cohort was characterized by middle-to-older age, early-stage disease, predominance of endometrioid histology, and a high BMI rate. These features are consistent with previously reported epidemiological patterns in EC. To ensure unbiased model training and evaluation, the dataset was randomly divided at the patient level into a training set (80%) and a test set (20%). Clinical and molecular subtype distributions were well balanced between the two sets, ensuring the robustness and generalizability of the model development and evaluation process (Table S2 and Table S3).

### Backbone model comparison for molecular subtype classification

To determine the most suitable network architecture for histology-based molecular subtype classification, multiple state-of-the-art deep learning backbones were systematically compared under identical preprocessing and training conditions. The architectures evaluated included EfficientNet, DenseNet, VGGNet, Vision Transformer (ViT), and ResNet-101, all tested on the independent internal validation set. Model performance was assessed using overall accuracy, sensitivity, specificity, and AUC.

**Fig. 2:**
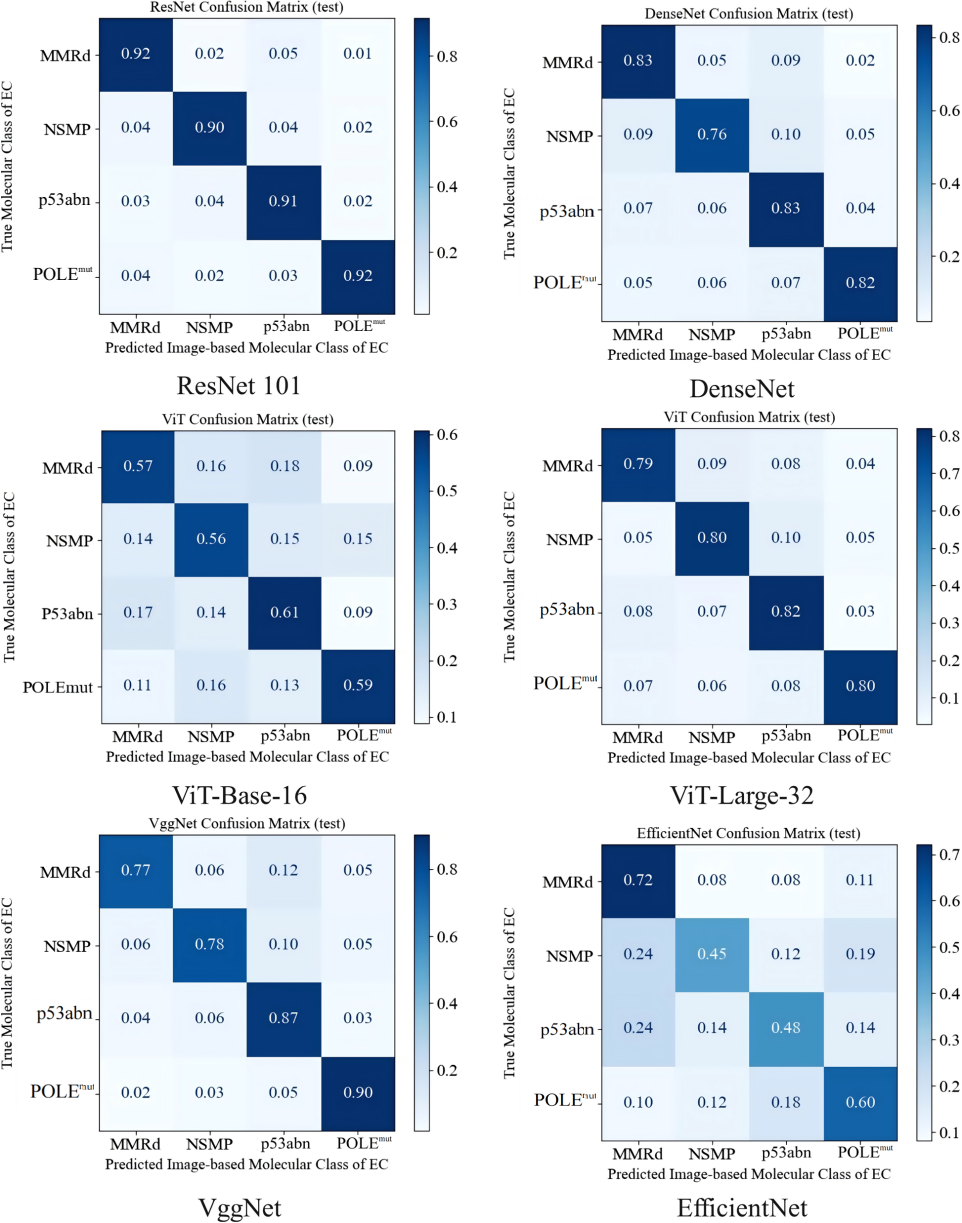
Confusion matrix of the classification model

Among these architectures, ResNet-101 demonstrated the most consistent and superior performance, achieving high classification accuracies of 92.0% for MMRd, 90.0% for NSMP, 91.0% for p53abn, and 92.0% for POLE^mut^ (Fig. 2). Other networks such as VGGNet and DenseNet achieved moderate yet competitive performance, whereas EfficientNet showed relatively lower accuracies across subtypes (Table 2).

**Table 2:** Accuracy Comparison of Image-Based Molecular Classification of EC

Model	MMRD AUC	NSMP AUC	p53abn AUC	POLE^mut^ AUC	Accuracy	Specificity	Sensitivity
Vgg-16	0.77	0.78	0.87	0.90	0.851	0.80053908	0.86902751
Vgg-19	0.89	0.87	0.86	0.94	0.887	0.8852941	0.8973226
EfficientNet	0.72	0.45	0.48	0.60	0.506	0.72131148	0.55296918
ViT-Base-16	0.57	0.56	0.61	0.59	0.619	0.58860759	0.58326841
ViT-Base-32	0.64	0.56	0.60	0.59	0.600	0.64359862	0.61093588
ViT-Large-32	0.79	0.80	0.82	0.80	0.859	0.82831325	0.82443111
DenseNet 161	0.83	0.76	0.83	0.82	0.820	0.84761905	0.81183389
ResNet-34	0.90	0.93	0.93	0.96	0.929	0.89944134	0.95092408
ResNet-50	0.93	0.90	0.91	0.97	0.926	0.90056818	0.93434032
ResNet 101	0.92	0.90	0.91	0.92	0.914	0.9244713	0.9073393

Collectively, These findings indicate that ResNet-101 provides the most reliable backbone for histology-based molecular subtype classification of endometrial cancer, combining strong discriminative power with balanced performance across all subtypes.

### Interpretation of AI-assisted molecular classification results

To clarify the rationale underlying the classification decisions, gradient-weighted class activation mapping (Grad-CAM) [14] and related interpretability methods were applied to highlight regions within images that influenced the deep learning model's predictions [15]. These techniques identified histological regions most closely associated with predicted molecular subtypes, allowing for visual assessment of the model's decision-making process. Grad-CAM generates class-discriminative localization maps, providing visual cues for model attention during subtype prediction. Grad-CAM was employed on the trained models to visualize the histological features utilized in classifying each case [16].

**Fig. 3:**
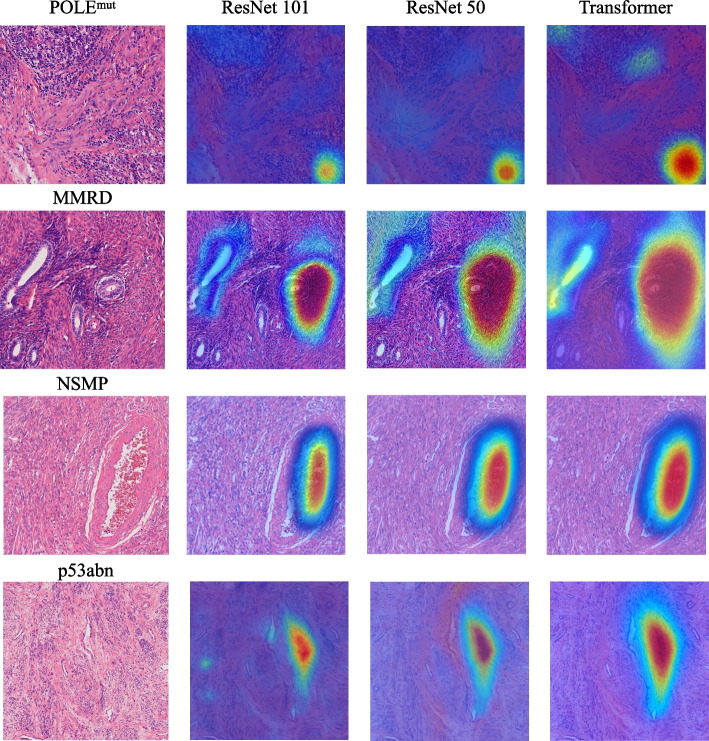
Comparative visualization of EC pathology images using diverse backbones. Each row corresponds to a distinct case within the dataset, showcasing the interpretative results. The visual comparison emphasizes the differences in diagnostic features captured by each backbone, providing insights into their respective performance in pathological image analysis

Grad-CAM heatmaps were generated from hematoxylin and eosin (HE)-stained WSIs using the trained ResNet-101 model and are presented in Fig. 3. For MMRd samples, the model primarily focused on glandular regions accompanied by dense immune cell infiltration at the tumor invasive front, consistent with the characteristic lymphocytic patterns often associated with MMRd tumors. In NSMP samples, attention was focused on well-preserved glandular morphology with mild nuclear atypia. For p53abn cases, the highlighted areas corresponded to regions with significant nuclear pleomorphism and prominent nuclear staining. In POLE^mut^ samples, the model emphasized areas with well-preserved glandular structures. It is important to note that these histological features are not definitive molecular diagnostic markers but represent statistically associated morphological characteristics identified by the model during training from large-scale data. Previous studies have also reported that deep learning models trained on HE-stained slides can accurately infer microsatellite instability (MSI), POLE mutations, and p53 abnormalities, validating the reliability of this interpretability framework [17–19].

### ROC analysis confirms high discriminative power of ResNet-101

**Fig. 4:**
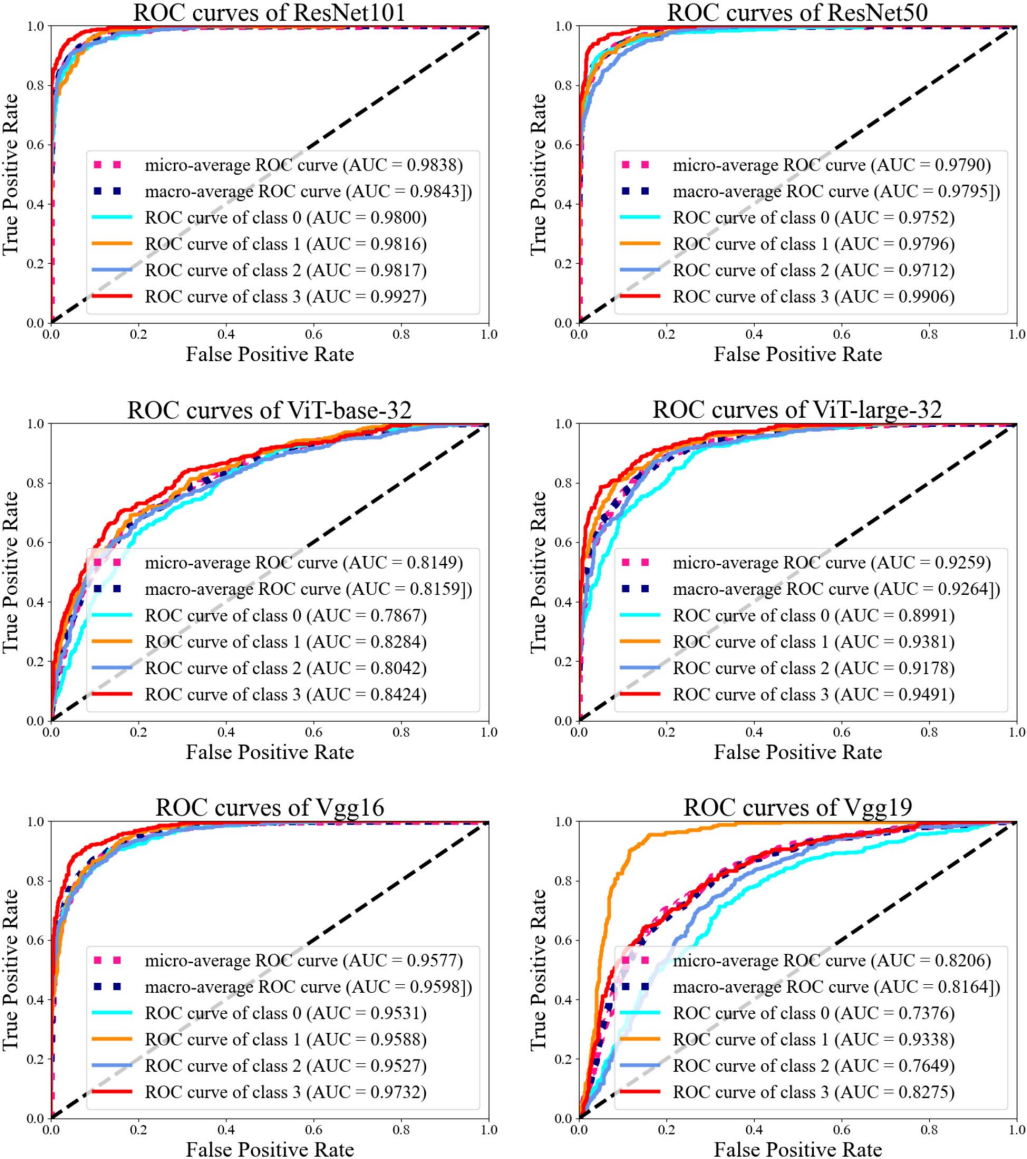
ROC curves and AUC of classification models. The y axis is the true positive rate, and the x axis is the false positive rate. ResNet-101 outperformed in most prediction tasks. Class 0 to Class 3 represent the four types of EC molecular classification from MMRD, NSMP, p53abn and POLEmut respectively

Receiver operating characteristic (ROC) analysis and corresponding area under the ROC curve (AUC) values were used to assess the ability of deep learning models to classify molecular subtypes of EC (Fig. 4). To address the class imbalance within the dataset, both micro-average and macro-average ROC curves were constructed for a comprehensive evaluation.

Among all architectures tested, ResNet-101 showed the strongest classification performance, achieving a micro-average AUC of 0.9838 (95% CI: 0.9768–0.9896) and a macro-average AUC of 0.9843 (95% CI: 0.9773–0.9903) (Table 2). Subtype-specific AUC values remained consistently high, ranging from 0.9800 for MMRd to 0.9927 for POLE^mut^. The model also maintained a balanced sensitivity of 90.7% and specificity of 92.4%, with an overall accuracy of 91.4%, and performed especially well for the POLE^mut^ subtype. ResNet-50 and ResNet-34 also performed well and achieved similar accuracies of 92.6% and 92.9%, respectively, but demonstrated slightly less balance between sensitivity and specificity compared to ResNet-101. Although ResNet-34 and ResNet-50 achieved overall accuracies close to ResNet-101, their macro-AUC values—particularly for minority subtypes such as POLE^mut^ and MMRd—were lower, reflecting weaker discrimination across thresholds. Therefore, backbone selection was guided primarily by AUC, while accuracy was retained as a secondary reference. VGG-16 and VGG-19 showed moderate performance and were particularly reliable for POLE^mut^ classification. DenseNet-161 achieved acceptable results but did not match the performance of the ResNet series. Transformer-based models (ViT-Base and ViT-Large) were less effective, with ViT-Large outperforming its Base counterpart but failing to exceed the convolutional neural network (CNN) models. EfficientNet showed the lowest performance across metrics, with an overall classification accuracy of approximately 50%.Across all models, the POLE^mut^ subtype consistently achieved the highest classification accuracy, which likely reflects its distinct morphological features. This may reflect distinct histological features, including increased lymphocytic infiltration and relatively preserved glandular architecture, which provide recognizable morphological cues for the model. These results indicate that ResNet-101, with the highest AUC and balanced sensitivity–specificity, effectively captures subtype-specific histological patterns and offers the most reliable performance for image-based molecular subtyping of EC.

### Survival prediction performance

The model demonstrated robust performance in estimating survival outcomes. Kaplan–Meier analysis based on ground-truth molecular classifications (Fig. S2a) revealed subtype-specific survival distributions, although statistical significance was not reached (Log-rank *P* = 0.18). Patients with POLE^mut^ tumors showed the most favorable long-term survival, in line with existing clinical literature, despite the limited sample size. The p53abn and MMRd subtypes were associated with less favorable outcomes. Survival curves based on model-predicted subtypes (Fig. S2b) were closely similar to the actual stratification, suggesting that the model successfully approximated biologically meaningful distinctions. All survival analyses are conducted on the independent test set only (n ≈ 80), defined a priori to avoid overlap with training and validation data. External validation cohorts do not include survival endpoints, and no survival modeling is performed on those cohorts.

To quantitatively evaluate the accuracy of survival time prediction, correlation analyses were conducted between actual and model-predicted durations (Fig. S2c). A strong linear association was observed, with Pearson, Spearman, and Kendall correlation coefficients of 0.9850, 0.9858, and 0.9036, respectively. The coefficient of determination (R2) reached 0.9692, indicating that the model explained 96.9% of the variance in observed survival durations. The mean absolute error (MAE) was 123.33 days, and the root mean squared error (RMSE) was 179.62 days, underscoring high precision in continuous outcome prediction. A Bland–Altman plot (Fig. S2d) further confirmed strong agreement between predicted and actual survival times, with the majority of points falling within the 95% limits of agreement (± 1.96 SD) and no signs of systematic deviation or variance instability.

In summary, although survival curves did not show statistically significant differences in this cohort, the model accurately reflected expected survival patterns and yielded precise predictions of individual survival durations. These findings underscore the potential of the model to support prognostic stratification and personalized treatment planning for patients with EC.

### External validation performance on multi-center cohorts

The ResNet-101 model demonstrated robust classification performance across two independent external validation cohorts. In the Obstetrics and Gynecology Hospital of Fudan University cohort (OGHFU, *N* = 83), it achieved an AUC of 0.97, with an overall accuracy of 92%, specificity of 94%, and sensitivity of 87%. In the Pingdingshan Maternal and Child Health Hospital cohort (PMCHH, *N* = 35), the AUC was 0.94, with an accuracy of 93%, specificity of 90%, and sensitivity of 94% (Fig. S3).

Compared to ResNet-50 and Transformer-based architectures, ResNet-101 achieved higher AUCs for all four molecular subtypes (POLE^mut^, MMRd, NSMP, and p53abn) across both centers. Despite inter-institutional variation in slide preparation and staining protocols, the model retained acceptable predictive performance, indicating potential applicability in diverse clinical settings. However, domain adaptation strategies may be required to further improve cross-center generalizability (Table 3 and Table 4).

**Table 3:** Baseline characteristics of the external validation cohorts (OGHFU and PMCHH)

Characteristic	OGHFU (*N* = 83)	PMCHH (*N* = 35)	*P*-value
Age group, n(%)			0.244
20–40 years	6 (7.23%)	2 (5.71%)	
40–50 years	19 (22.89%)	9 (25.71%)	
50–70 years	31 (37.35%)	7 (20.00%)	
70 + years	27 (32.53%)	17 (48.57%)	
Molecular classification, n(%)			0.758
MMRd	19 (22.89%)	8 (22.86%)	
NSMP	43 (51.81%)	20 (57.14%)	
p53abn	12 (14.46%)	4 (11.43%)	
POLE^mut^	13 (15.67%)	3 (8.57%)	
Histological type, n(%)			0.010
Carcinosarcomas	1 (1.20%)	0 (0%)	
Clear Cell Carcinoma	0 (0%)	1 (2.86%)	
Endometrioid Carcinoma G1	60 (72.29%)	15 (42.86%)	
Endometrioid Carcinoma G2	5 (6.02%)	8 (22.86%)	
Endometrioid Carcinoma G3	11 (13.25%)	7 (20.00%)	
Mixed Carcinoma	0	2 (5.71%)	
Serous Carcinoma	5 (6.02%)	2 (5.71%)	
Undifferentiated Carcinoma	1 (1.20%)	0 (0%)	
Stage, n(%)			0.698
I	62 (74.70%)	24 (68.57%)	
II	10 (12.05%)	7 (20.00%)	
III	7 (8.43%)	3 (8.57%)	
IV	4 (4.82%)	1 (2.86%)

**Table 4:** Performance of different backbones on external validation cohorts (OGHFU and PMCHH)

Cohort	Model	MMRD AUC	NSMP AUC	p53abn AUC	POLE^mut^ AUC	Accuracy	Specificity	Sensitivity
OGHFU	ResNet-50	0.93	0.91	0.94	0.96	0.88	0.87	0.83
	ResNet-101	0.96	0.94	0.97	0.98	0.92	0.94	0.87
	Transformer	0.84	0.92	0.95	0.94	0.91	0.91	0.85
PMCHH	ResNet-50	0.90	0.88	0.89	0.94	0.89	0.84	0.88
	ResNet-101	0.93	0.91	0.94	0.97	0.93	0.90	0.94
	Transformer	0.91	0.89	0.92	0.96	0.90	0.90	0.88

## Discussion

In this retrospective study, approximately 30% of patients with EC were aged between 20 and 50 years, indicating a trend toward earlier onset. This demographic shift highlights the clinical need for personalized therapeutic strategies, particularly in patients seeking fertility preservation. With the increasing volume of pathological specimens, the demand for accurate and timely histological evaluation has increased, while the global shortage of trained pathologists remains unresolved. To address this challenge, AI-assisted digital pathology has emerged as a potential solution, offering reproducible, high-throughput analysis with the ability to detect subtle histological features, improving diagnostic consistency and efficiency.

Given the high cost, procedural complexity, and long turnaround time of POLE mutation testing and IHC analysis, this study proposes a practical, image-based alternative for molecular classification of EC. Using WSIs and a deep learning framework, the approach provides a noninvasive and scalable solution for both molecular subtyping and prognostic estimation. Importantly, this pipeline could facilitate timely molecular characterization for patients undergoing fertility-preserving management or for institutions with limited access to molecular testing, thereby bridging the gap between precision oncology and routine diagnostic pathology. Compared with IHC and NGS/Sanger sequencing, which often require several days to weeks and specialized laboratory personnel, the proposed AI pipeline can provide preliminary subtype predictions within hours once WSIs are available, reducing turnaround time and manpower demand. In resource-limited settings, this approach may serve as a cost-efficient, complementary screening layer to prioritize confirmatory molecular testing rather than replacing existing diagnostic workflows.

The analysis workflow incorporated super-resolution enhancement via SRResGAN and precise lesion segmentation using MedSAM, followed by model training with multiple neural network architectures. Among the evaluated models, ResNet-101 showed the highest classification accuracy for all four molecular subtypes, achieving over 90% classification accuracy. Grad-CAM visualizations confirmed that the model's focused regions corresponded with histopathological features consistent with known subtype characteristics, suggesting that morphological signals captured in routine HE-stained slides are predictive of underlying molecular alterations. These findings demonstrate that deep learning models can infer clinically relevant molecular profiles directly from histological images, enabling integration of molecular subtyping and prognosis estimation from a single modality. The model's classification performance was further validated using two external, multi-center, independent cohorts. The stable classification accuracy across centers with different data sources suggests generalizability and supports the potential applicability of this pipeline in real-world clinical practice. Despite differences in slide preparation and imaging equipment between institutions, model performance remained stable, indicating resistance to domain-specific variation. Model performance may vary across histologic subtypes, stages, and age groups. As the current dataset consisted predominantly of endometrioid ECs, caution is warranted when interpreting predictions for rare histologic variants or specimens with low tumor cellularity, in which morphological cues may be limited. Moreover, fertility-sparing and younger patients represent important subgroups for future validation, as hormonal status or sampling differences could influence image-based classification. These clarifications define the scope of applicability and support cautious clinical translation of the model. Previous AI-assisted molecular subtyping studies, including the TCGA-based work by Frémond et al. [13], were primarily developed and validated on research datasets. By contrast, the present study leverages large, multi-center Chinese clinical cohorts derived from routine diagnostic practice, providing real-world evidence of the feasibility and robustness of AI-based molecular subtyping beyond TCGA-derived settings. Although SRResGAN can enhance fine-grained structures that assist ROI delineation, it may also introduce artifacts; the pipeline therefore prioritizes a pragmatic trade-off by limiting SR intensity and relying on augmentation-based robustness, while acknowledging that future evaluations should compare explicit stain normalization and SR-free baselines. The modest size of our internal test set, coupled with the limited scale of the external validation cohorts (*N* = 35 and *N* = 83), inherently constrains the statistical power of our analysis. Consequently, the reported performance metrics, including the AUCs, should be interpreted with caution. Our findings are therefore presented as preliminary, and the generalizability of our model remains a hypothesis that warrants confirmation through larger, prospectively accrued, and multi-regional studies.

Although Kaplan–Meier survival analysis did not show statistically significant differences across molecular subtypes, patients with POLE^mut^ displayed more favorable long-term survival trends, consistent with previous clinical observations. The lack of statistical significance may be due to the limited number of events and relatively short follow-up duration in this subgroup. Overall, most patients in this cohort remained alive during follow-up, supporting the reliability of the survival estimates and aligning with the typical prognosis of early-stage EC.

The model predicts molecular subtypes defined by IHC and Sanger sequencing, not prognosis itself. The subsequent survival analysis should be regarded as exploratory, serving as proof-of-concept that image-based molecular predictions may correlate with patient outcomes. These findings are preliminary and not intended as formal prognostic validation. In practical deployment, each model output is accompanied by a confidence score. Low-confidence or discordant predictions should trigger confirmatory molecular testing or multidisciplinary review before informing clinical management. Acceptable risk thresholds may differ across clinical scenarios, with stricter confidence requirements for therapeutic decision-making than for triage or screening applications.

This study has several limitations that should be acknowledged. First, cause-specific mortality was not assessed, and deaths unrelated to EC may have introduced bias. Second, while patients with other malignancies were excluded, the presence of baseline comorbidities was not analyzed, potentially introducing confounding effects. Third, although the model effectively predicted molecular subtypes and survival durations, clinical covariates were not included in the survival prediction module, limiting its ability to account for non-histological prognostic factors. Additionally, the retrospective design, limited follow-up duration, and exclusion of cases with incomplete or poor-quality data may have introduced selection bias, and all participating centers were Chinese institutions, which could limit international generalizability. These factors should be considered when interpreting the results, which are intended to be hypothesis-generating rather than definitive. Incorporating such data in future iterations may improve predictive accuracy and clinical relevance. Future work will also focus on integrating WSI-derived features with key clinical covariates and IHC metadata to build calibrated multivariable prognostic models; extending and adapting the pipeline to related tumor types (e.g., cervical, ovarian, colorectal) within prospective, multi-regional cohorts; enhancing transportability through stain handling and domain adaptation; and exploring histopathology-native pretraining with human-in-the-loop workflows to support safe clinical deployment.

In summary, this study evaluated the feasibility of applying deep learning to WSIs for molecular subtyping and survival prediction in EC. To our knowledge, this represents the first AI-based, large-scale multi-center analysis within a Chinese population, confirming that histology-based models can be used to classify the four TCGA molecular subtypes of EC with high accuracy (> 90%) without the need for molecular testing and IHC. The results support the use of digital pathology combined with AI to provide a low-cost, scalable approach to guide individualized clinical management and improve prognostic assessment in patients with EC.

## Materials and methods

### Study design and participants

This retrospective cohort study was conducted at Shanghai First Maternal and Infant Hospital and included patients newly diagnosed with EC between January 2010 and December 2018. Patients with a history of other malignancies or previous treatment for EC were excluded, resulting in an initial cohort of 444 patients. All diagnoses and treatment decisions were made by attending physicians based on European Society for Medical Oncology (ESMO) guidelines and patient preferences [20, 21]. Clinical and pathological data were retrieved from archived medical records, and annual follow-up was conducted via telephone. After excluding 22 patients with incomplete clinical or pathological information, 422 cases remained. The other 13 were excluded due to poor slide quality, 1 due to an unclassifiable molecular subtype, and 15 due to missing key molecular data. Finally, 393 patients with complete molecular subtyping and survival data were included in the analysis.

The resulting dataset comprised 15,328 histopathological images from these 393 patients, classified into four molecular subtypes: POLE^mut^ (*N* = 45), MMRd (*N* = 73), p53abn (*N* = 115), and NSMP (*N* = 160). Tumor regions were delineated using a finely tuned MedSAM model [22], and image patches were then extracted. The dataset was randomly divided into training and testing sets in an 8:2 ratio, ensuring that image data from the same patient were assigned exclusively to a single subset to avoid data leakage.

To evaluate external validity, two independent validation cohorts were additionally collected from the OGHFU (*N* = 83) and PMCHH (*N* = 35). These datasets were prepared under routine clinical workflows at each institution, scanned with locally available digital pathology systems, and labeled according to the same four molecular subtypes. No external samples were used during model training (Fig. S1).

### Tissue processing and digital slide acquisition

Tissue specimens obtained from hysterectomy were immediately fixed in 4.7% aldehyde solution at room temperature for at least 4 h. Following HE staining, WSIs were acquired using a high-resolution scanner at 20 × magnification. Pathological sections from 444 patients underwent POLE mutation testing and IHC analysis for markers including PMS2, MLH1, MSH2, MSH6, and P53 [23]. Digital images were generated using Digital Slide Assistant Lite and converted to a standardized format using OpenSlide. All digital slides were reviewed by experienced pathologists.

### Digital pathology image preprocessing procedure

The preprocessing pipeline began with image enhancement using the SRResGAN model [24], which transforms low-resolution WSIs into high-resolution images. This enhancement was essential for capturing microstructural tissue features critical to histopathological evaluation. High-resolution images offer the detail needed to identify and study subtle morphological differences, which are crucial for accurate diagnosis and subtype classification [25]. SRResGAN, a generative adversarial network pre-trained on ImageNet and COCO datasets, was employed to increase image resolution while preserving feature integrity across different scales. The model minimized oversmoothing artefacts that may hide or distort diagnostically relevant structures. Enhanced image resolution facilitated both pathologists and AI models to make more precise assessments by providing clearer, more detailed visual information, supporting improved detection and analysis of histopathological features [26]. To reduce the risk of hallucination or morphology exaggeration from SRResGAN, a conservative configuration was used (limited upscaling and suppressed edge sharpening) and applied uniformly as a preprocessing step prior to segmentation/classification; robustness to potential domain shift was addressed with color/stain and scanner augmentations. No explicit color normalization was applied in the primary pipeline; instead, robustness to inter-laboratory staining and scanner variability was addressed via augmentation during training.

### Targeted segmentation of cancerous regions

To selectively identify and outline EC lesions within WSIs, the MedSAM model [27] was employed. This segmentation method uses the synergy between visual and prompt-based features, further refined through image enhancement techniques. Accurate definition of ROIs facilitates concentration of computational resources on diagnostically relevant areas [25], reducing analytical burden and improving the precision of subsequent analysis. MedSAM employs a transformer-based framework to encode visual features from pathology slides, initialized with pretrained weights that capture generic structural patterns from large-scale image datasets. These pretrained weights facilitate the recognition of basic image elements such as edges, lines, textures, and contours, which form the solid foundation for task-specific refinement tailored to EC lesion segmentation. The model also integrates a prompt-based technique, wherein bounding boxes are used to direct the segmentation task toward defined ROIs, specifically annotated as "endometrial cancer lesions. This multimodal architecture allows MedSAM to accurately segment regions likely to harbor EC pathology, typically appearing as eosinophilic tumor areas with darker nuclei against a paler stromal background of normal endometrial tissue [28]. The segmentation output is a pixel-wise segmentation mask that outlines cancerous areas within each WSI. This targeted segmentation both improves computational efficiency by omitting irrelevant regions and provides a spatially resolved representation of lesion morphology and architecture, which are essential for further diagnostic evaluations. ROI segmentation was configured to balance noise reduction with preservation of tumor–microenvironment context. Masks were generated with conservative margins around tumor regions so that peri-tumoral stroma, invasion fronts, and immune-cell–rich areas remained within the analysis field, while excluding obviously irrelevant background. This design aims to retain architectural and immune cues that are informative for subtypes such as MMRd, thereby mitigating boundary effects introduced by tight croppin.

### Model development and training for molecular subtype classification

To achieve precise molecular classification of EC into four subtypes, a CNN architecture based on ResNet-101 was employed [29]. This model was trained on enhanced and segmented image patches sized 224 × 224 pixels, processed as multi-channel inputs to preserve spatial and contextual relationships. The network's 101-layer depth allowed for hierarchical integration of local visual features across a receptive field of 427 × 427 pixels, capturing broader architectural patterns in the tumor microenvironment. The ResNet-101 model incorporates residual connections with identity mappings to facilitate gradient flow during training and address vanishing gradient issues. It comprises 33 bottleneck blocks, each containing three convolutional layers with shortcut connections, enabling simultaneous spatial dimension reduction and feature channel expansion. This architecture allows the network to extract high-level abstract features from histopathological features [30].

The ResNet-101 backbone network was initialized with weights pretrained on the ImageNet dataset, which, despite being based on natural images instead of histopathology slides, provides a robust foundation for visual feature extraction. Transfer learning, widely used in medical imaging, was then applied by fine-tuning the model using histopathological images, allowing domain-specific adaptation of the extracted features to the task of EC molecular subtyping. Following feature extraction, global average pooling was applied, and the resultant representations were passed through two fully connected layers with 512 and 4 neurons, respectively. The final classification layer employed SoftMax activation to generate probability outputs corresponding to the four molecular subtypes: POLE^mut^, MMRd, p53abn, and NSMP. The training pipeline was optimized to ensure convergence and stability, supporting accurate classification of subtype-specific morphological features embedded within the pathology images. Patch-level posterior probabilities for each subtype are first computed by the classifier on tiled regions. For each slide, patch posteriors are aggregated by the mean of the per-patch probabilities to obtain slide-level subtype probabilities; the slide label is taken as the argmax of these aggregated probabilities. Patient-level decisions are then formed by aggregating across all slides from the same patient using a majority vote over slide labels, with a tie-breaker based on the highest mean slide-level probability. In addition to the final label, uncertainty is summarized by the dispersion of patch- and slide-level predictions and by inter-slide disagreement, enabling a quantitative confidence score at the patient level.

### Progression and survival prediction model

In the final step of the analytical pipeline, molecular subtype predictions generated by the deep learning model were integrated into a long short-term memory (LSTM) network to predict survival in EC patients. These subtype predictions served as input features alongside longitudinal clinical variables. The LSTM model was trained using the binary cross-entropy loss function and optimized with the Adam algorithm. Input data included annually collected sequential clinical parameters such as age at diagnosis, BMI, histological type, and predicted molecular subtype. Before entering the model, these variables were standardized. Sequence lengths varied depending on the duration and availability of follow-up records for each patient [31, 32].

This integrated approach, combining deep learning-based molecular classification from digital pathology images with LSTM-based temporal modeling of clinical parameters, aims to deliver a personalized and comprehensive risk assessment framework for EC patients.

### Evaluation metrics

Model performance across image preprocessing and classification stages was evaluated using standard metrics that assess both accuracy and robustness in identifying and classifying pathological features [33]. Key indicators included precision, recall, and AUC. To ensure objective assessment, all evaluations were conducted in a blinded manner; reviewers were unaware of model-generated predictions. Macro-averaged AUC was prespecified as the primary performance metric because it is threshold-independent and prevalence-invariant, which makes it more reliable under class imbalance. Accuracy, balanced accuracy, F1-score, and calibration error were reported as secondary measures to provide complementary perspectives.

For survival prediction, multiple quantitative metrics were applied to assess the concordance and accuracy between predicted and actual survival durations. These included Pearson, Spearman, and Kendall correlation coefficients, the coefficient of determination (R2), MAE, and RMSE, providing a comprehensive evaluation of predictive performance.

### Statistical analyses

All histological classifications, molecular subtyping, and staging were performed by experienced pathologists. Descriptive statistical comparisons among groups were primarily conducted using the Pearson chi-square test. Where appropriate, contingency table chi-square tests were applied to assess differences among categorical variables. Nonparametric comparisons were performed using the Kruskal–Wallis rank-sum test, Z-score test, and likelihood ratio test, as applicable. All analyses were conducted using R statistical software (version 4.3.1). Due to the limited number of patients with incomplete data and the negligible impact on the final results, these cases were excluded from the analysis.

### Model configuration

All models were developed using Python 3.9, PyTorch v1.12, and the AutoDL platform. Both the SRResGAN and classification model were trained for 200 epochs with a batch size of 32, using two NVIDIA A100 GPUs. Hyperparameter settings included an initial learning rate of 0.001, a final learning rate of 0.1 under the cosine annealing learning rate scheduler, a weight decay of 1e − 4, and a momentum coefficient of 0.9.

The LSTM-based survival prediction model employed a hidden layer comprising 1000 units and a dropout rate of 0.001 to reduce overfitting. This was followed by a dense output layer with a single neuron and a sigmoid activation function to yield survival probabilities between 0 and 1 for individual patients. The network was trained for 100 epochs with a batch size of 32 samples.

Key hyperparameters, including learning rate, batch size, and dropout rate, were selected through a limited grid search on the validation subset to balance convergence stability, computational efficiency, and generalization. The learning rate schedule (cosine annealing) ensured smooth decay to prevent divergence, while the batch size of 32 was constrained by GPU memory and maintained stable gradient estimation. A small dropout rate (0.001) was used to regularize the fully connected layers without impairing convergence.

### Vision Transformer (ViT) Implementation

To benchmark the classification model performance, a ViT architecture was also evaluated. The ViT model was pretrained on the ImageNet dataset and then fine-tuned using the same histopathology image patches and training conditions as those applied for CNN-based model.

## Supplementary Information

Supplementary Material 1.

## Data Availability

The corresponding author will provide the original data used to support the findings of this study upon reasonable request.
