# Supplementary material for: Clinical-grade AI model for molecular subtyping of endometrial cancer: a multi-center cohort study in China
Source: Mol Biomed. 2025 Nov 10;6:102. doi: 10.1186/s43556-025-00341-z (PMC12597869; doi:10.1186/s43556-025-00341-z)
Supplement: Supplementary file 1 — Supplementary Material 1. [file 43556_2025_341_MOESM1_ESM.docx]

**Clinical-grade AI model for molecular subtyping of endometrial cancer: A multi-center cohort study in China**

Peng Qi^1,6†^, Tianliang Yao^1†^, Hu Li^2†^, Jingnan Zhu^2†^,Jianye Li^3^, Xuezhen Luo^4*^, Qizhi He^5*^, Yiran Li^2*^

^1^Department of Control Science and Engineering, College of Electronics and Information Engineering, Tongji University, Shanghai, 201210, China.

^2^Centre for Assisted Reproduction, Shanghai Key Laboratory of Maternal-Fetal Medicine, Shanghai Institute of Maternal-Fetal Medicine and Gynecologic Oncology, Shanghai First Maternity and Infant Hospital, School of Medicine, Tongji University, Shanghai, 200092, China.

^3^Pingdingshan Women and Children Health Care Hospital, Henan, 467000, China.

^4^Department of Gynecology, Obstetrics and Gynecology Hospital of Fudan University, Shanghai, 200011, China.

^5^Department of Pathology, Shanghai First Maternity and Infant Hospital, School of Medicine, Tongji University, Shanghai, 200092, China.

^6^State Key Laboratory of Cardiovascular Diseases and Medical Innovation Center, Shanghai East Hospital, School of Medicine, Tongji University, Shanghai, 200092, China.

*Correspondence:

xuezhenluo2013@163.com; qizhihe@tongji.edu.cn; liyiran2007@gmail.com;

^†^Peng Qi, Tianliang Yao, Hu Li and Jingnan Zhu contributed equally to this work.

**Figure S1.** Flow diagram illustrating the inclusion and exclusion of patients and the construction of the study cohorts.

A total of 444 patients were initially evaluated, of whom 51 were excluded due to incomplete clinical or pathological information, poor slide quality, uncertain molecular classification, or missing molecular data. The remaining 393 patients were included and randomly divided into a training cohort (n = 313) and a test cohort (n = 80). Two independent external cohorts (OGHFU, n = 83; PMCHH, n = 35) were used for validation.


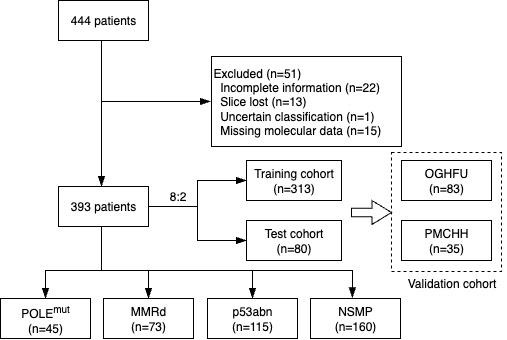


**Figure S2.** Survival analysis and performance evaluation of the predictive model.

(a). Kaplan–Meier survival curves based on ground-truth molecular classifications of endometrial cancer patients.

(b). Kaplan–Meier survival curves based on model-predicted molecular classifications.

(c). Scatter plot showing the correlation between actual and predicted survival times; the red regression line indicates the overall fit.

(d). Bland–Altman plot showing the agreement between actual and predicted survival times, with most differences falling within the 95% limits of agreement.


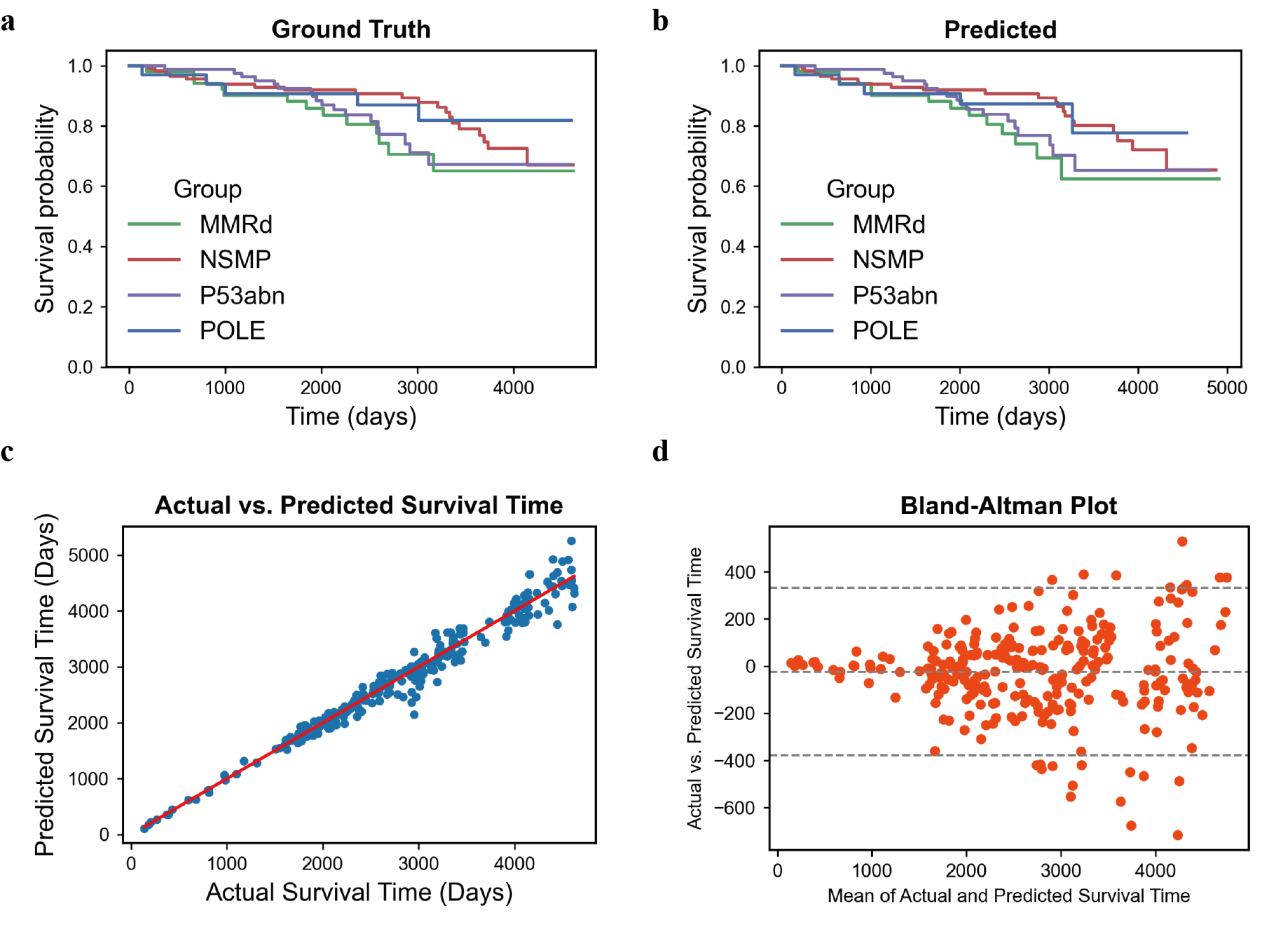


**Figure S3.** ROC curves of the ResNet‑101 model on external validation cohorts.

ROC curves for the ResNet‑101 model on two independent cohorts: OGHFU (AUC = 0.97) and PMCHH (AUC = 0.94).


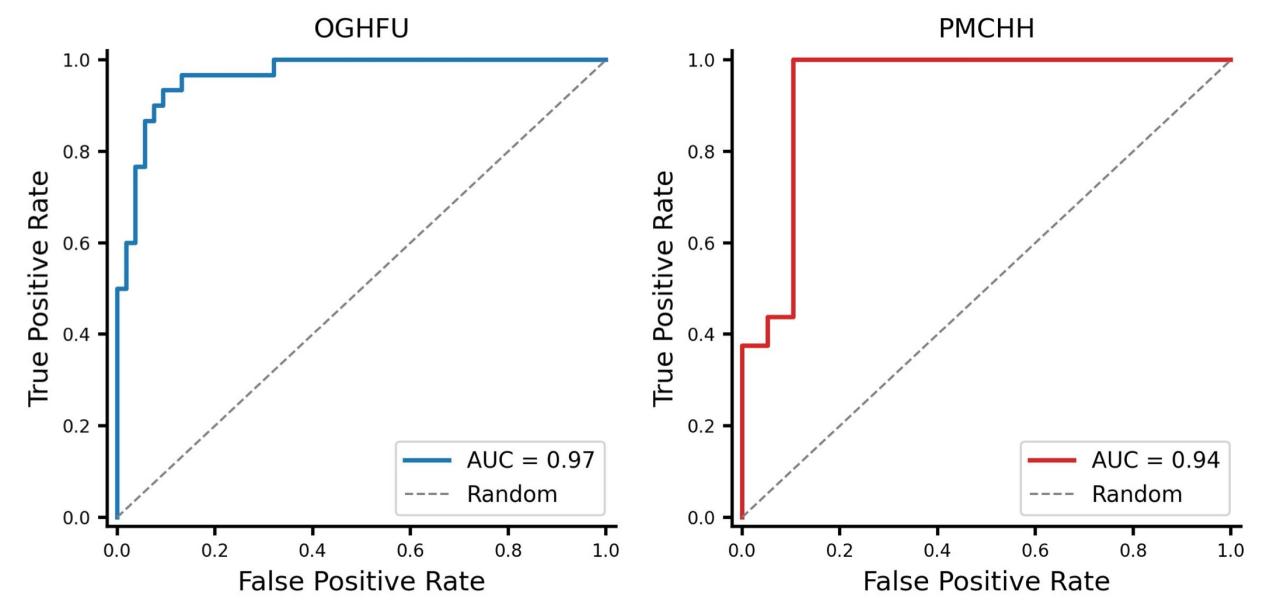


**Table S1**. Patient characteristics by molecular classification.

| Characteristic | MMRd (N= 73) | NSMP (N = 160) | p53abn (N = 115) | POLE^mut^ (N = 45) | P-value |
| --- | --- | --- | --- | --- | --- |
| Death, n(%) |  |  |  |  | 0.145 |
| Alive | 56 (76.71%) | 133 (83.13%) | 87 (75.65%) | 39 (86.67%) |  |
| Dead | 11 (15.07%) | 12 (7.5%) | 18 (15.65%) | 6 (13.33%) |  |
| Lost | 6 (8.22%) | 15 (9.37%) | 10 (8.7%) | 0 (0%) |  |
| Age group, n(%) |  |  |  |  | 0.122 |
| 20-40 years | 1 (1.37%) | 11 (6.88%) | 3 (2.61%) | 3 (6.67%) |  |
| 40-50 years | 19 (26.03%) | 46 (28.75%) | 28 (24.35%) | 6 (13.33%) |  |
| 50-70 years | 48 (65.75%) | 97 (60.63%) | 80 (69.57%) | 36 (80%) |  |
| 70+ years | 5 (6.85%) | 6 (3.75%) | 4 (3.48%) | 0 (0%) |  |
| Histological type, n(%) |  |  |  |  | 0.503 |
| Carcinosarcomas | 3 (4.11%) | 6 (3.75%) | 4 (3.48%) | 1 (2.22%) |  |
| Clear Cell Carcinoma | 0 (0%) | 2 (1.25%) | 2 (1.74%) | 1 (2.22%) |  |
| Endometrioid Carcinoma G1 | 50 (68.49%) | 117 (73.13%) | 83 (72.17%) | 25 (55.56%) |  |
| Endometrioid Carcinoma G2 | 10 (13.70%) | 17 (10.63%) | 12 (10.43%) | 10 (22.22%) |  |
| Endometrioid Carcinoma G3 | 4 (5.48%) | 3 (1.88%) | 1 (0.87%) | 2 (4.44%) |  |
| Mixed Carcinoma | 2 (2.74%) | 2 (1.25%) | 4 (3.48%) | 1 (2.22%) |  |
| Serous Carcinoma | 3 (4.11%) | 13 (8.13%) | 9 (7.83%) | 5 (11.11%) |  |
| Undifferentiated Carcinoma | 1 (1.37%) | 0 (0%) | 0 (0%) | 0 (0%) |  |
| Stage, n(%) |  |  |  |  | 0.010 |
| I | 59 (80.82%) | 117 (73.13%) | 70 (60.87%) | 39 (86.67%) |  |
| II | 9 (12.33%) | 21 (13.13%) | 18 (15.65%) | 4 (8.89%) |  |
| III | 4 (5.48%) | 21 (13.13%) | 27 (23.48%) | 2 (4.44%) |  |
| IV | 1 (1.37%) | 1 (0.63%) | 0 (0%)\ | 0 (0%) |  |
| BMI group |  |  |  |  | 0.804 |
| Under weight | 4 (5.48%) | 5 (3.12%) | 2 (1.74%) | 1 (2.22%) |  |
| Normal weight | 15 (20.55%) | 40 (25.00%) | 23 (20.00%) | 7 (15.56%) |  |
| Over weight | 33 (45.21%) | 75 (46.88%) | 58 (50.43%) | 22 (48.89%) |  |
| Obese | 22 (30.14%) | 40 (25.00%) | 40 (27.83%) | 15 (33.33%) |  |

**Table S2**. Baseline characteristics of training set.

| Characteristic | MMRd (N = 55) | NSMP (N = 136) | p53abn (N = 84) | POLE^mut^ (N = 38) | P-value |
| --- | --- | --- | --- | --- | --- |
| Death |  |  |  |  | 0.1216 |
| alive | 43 (78.2%) | 114 (83.8%) | 61 (72.6%) | 34 (89.5%) |  |
| death | 8 (14.5%) | 10 (7.4%) | 15 (17.9%) | 4 (10.5%) |  |
| lost | 4 (7.3%) | 12 (8.8%) | 8 (9.5%) | 0 (0%) |  |
| Age group |  |  |  |  | 0.136 |
| 20-40 | 1 (1.82%) | 9 (16.36%) | 2 (2.38%) | 2 (5.26%) |  |
| 40-50 | 14 (25.45%) | 39 (28.68%) | 20 (23.81%) | 5 (13.16%) |  |
| 50-70 | 36 (65.45%) | 83 (61.03%) | 61 (72.62%) | 31 (81.58%) |  |
| 70+ | 4 (7.27%) | 5 (3.68%) | 1 (1.19%) | 0 (0.00%) |  |
| Histologicaltype |  |  |  |  | 0.857 |
| Carcinosarcomas | 2 (3.64%) | 5 (3.68%) | 3 (3.57%) | 1 (2.63%) |  |
| Clear Cell Carcinoma | 0 (0.00%) | 2 (1.47%) | 1 (1.19%) | 1 (2.63%) |  |
| Endometrioid Carcinoma G1 | 37 (67.27%) | 99 (72.79%) | 61 (72.62%) | 21 (55.26%) |  |
| Endometrioid Carcinoma G2 | 8 (14.55%) | 15 (11.03%) | 9 (10.71%) | 8 (21.05%) |  |
| Endometrioid Carcinoma G3 | 3 (5.45%) | 2 (1.47%) | 1 (1.19%) | 2 (5.26%) |  |
| Mixed Carcinoma | 2 (3.64%) | 2 (1.47%) | 3 (3.57%) | 1 (2.63%) |  |
| Serous Carcinoma | 3 (5.45%) | 11 (8.09%) | 6 (7.14%) | 4 (10.53%) |  |
| Undifferentiated Carcinoma | 0 (0.00%) | 0 (0.00%) | 0 (0.00%) | 0 (0.00%) |  |
| Stage |  |  |  |  | 0.045 |
| I | 44 (80.00%) | 99 (72.79%) | 51 (60.71%) | 33 (86.84%) |  |
| II | 7 (12.73%) | 18 (13.24%) | 13 (15.48%) | 3 (7.89%) |  |
| III | 3 (5.45%) | 18 (13.24%) | 20 (23.81%) | 2 (5.26%) |  |
| IV | 1 (1.82%) | 1 (0.74%) | 0 (0.00%) | 0 (0.00%) |  |
| BMI |  |  |  |  | 0.929 |
| Under weight | 3 (5.45%) | 4 (2.94%) | 2 (2.38%) | 1 (2.63%) |  |
| Normal weight | 11 (20.00%) | 34 (25.00%) | 17 (20.24%) | 6 (15.79%) |  |
| Over weight | 25 (45.45%) | 64 (47.06%) | 42 (50.00%) | 18 (47.37%) |  |
| Obese | 16 (29.09%) | 34 (25.00%) | 23 (27.38%) | 13 (34.21%) |  |

**Table S3**. Baseline characteristics of test set.

| Characteristic | MMRd (N = 18) | NSMP (N = 24) | P53abn (N = 31) | POLE-mut (N = 7) | P-value |
| --- | --- | --- | --- | --- | --- |
| Death |  |  |  |  | 0.707 |
| alive | 13 (72.2%) | 19 (79.2%) | 26 (83.9%) | 5 (71.4%) |  |
| death | 3 (16.7%) | 2 (8.3%) | 3 (9.7%) | 2 (28.6%) |  |
| lost | 2 (11.1%) | 3 (12.5%) | 2 (6.4%) | 0 (0%) |  |
| Age group |  |  |  |  | 0.857 |
| 20-40 | 0 (0.00%) | 2 (8.33%) | 1 (3.23%) | 1 (14.29%) |  |
| 40-50 | 5 (27.78%) | 7 (29.17%) | 8 (25.81%) | 1 (14.29%) |  |
| 50-70 | 12 (66.67%) | 14 (58.33%) | 19 (61.29%) | 5 (71.43%) |  |
| 70+ | 1 (5.56%) | 1 (4.17%) | 3 (9.68%) | 0 (0.00%) |  |
| Histologicaltype |  |  |  |  | 0.897 |
| Carcinosarcomas | 1 (5.56%) | 1 (4.17%) | 1 (3.23%) | 0 (0.00%) |  |
| Clear Cell Carcinoma | 0 (0.00%) | 0 (0.00%) | 1 (3.23%) | 0 (0.00%) |  |
| Endometrioid Carcinoma G1 | 13 (72.22%) | 18 (75.00%) | 22 (70.97%) | 4 (57.14%) |  |
| Endometrioid Carcinoma G2 | 2 (11.11%) | 2 (8.33%) | 3 (9.68%) | 2 (28.57%) |  |
| Endometrioid Carcinoma G3 | 1 (5.56%) | 1 (4.17%) | 0 (0.00%) | 0 (0.00%) |  |
| Mixed Carcinoma | 0 (0.00%) | 0 (0.00%) | 1 (3.23%) | 0 (0.00%) |  |
| Serous Carcinoma | 0 (0.00%) | 2 (8.33%) | 3 (9.68%) | 1 (14.29%) |  |
| Undifferentiated Carcinoma | 1 (5.56%) | 0 (0.00%) | 0 (0.00%) | 0 (0.00%) |  |
| Stage |  |  |  |  | 0.558 |
| I | 15 (83.33%) | 18 (75.00%) | 19 (61.29%) | 6 (85.71%) |  |
| II | 2 (11.11%) | 3 (12.50%) | 5 (16.13%) | 1 (14.29%) |  |
| III | 1 (5.56%) | 3 (12.50%) | 7 (22.58%) | 0 (0.00%) |  |
| BMI |  |  |  |  | 0.978 |
| Under weight | 1 (5.56%) | 1 (4.17%) | 0 (0.00%) | 0 (0.00%) |  |
| Normal weight | 4 (22.22%) | 6 (25.00%) | 6 (19.35%) | 1 (14.29%) |  |
| Over weight | 8 (44.44%) | 11 (45.83%) | 16 (51.61%) | 4 (57.14%) |  |
| Obese | 5 (27.78%) | 6 (25.00%) | 9 (29.03%) | 2 (28.57%) |  |
